# A fluorescence-based reporter for monitoring expression of mycobacterial cytochrome *bd* in response to antibacterials and during infection

**DOI:** 10.1038/s41598-017-10944-4

**Published:** 2017-09-06

**Authors:** Maikel Boot, Kin Ki Jim, Ting Liu, Susanna Commandeur, Ping Lu, Theo Verboom, Holger Lill, Wilbert Bitter, Dirk Bald

**Affiliations:** 1 Department of Medical Microbiology and Infection Control, VU University Medical Center, De Boelelaan 1108, 1081 HZ Amsterdam, The Netherlands; 2 Department of Molecular Cell Biology, Amsterdam Institute for Molecules, Medicines and Systems, Faculty of Earth- and Life Sciences, Vrije Universiteit Amsterdam, De Boelelaan 1108, 1081 HZ Amsterdam, The Netherlands

## Abstract

Cytochrome *bd* is a component of the oxidative phosphorylation pathway in many Gram-positive and Gram-negative bacteria. Next to its role as a terminal oxidase in the respiratory chain this enzyme plays an important role as a survival factor in the bacterial stress response. In *Mycobacterium tuberculosis* and related mycobacterial strains, cytochrome *bd* is an important component of the defense system against antibacterial drugs. In this report we describe and evaluate an mCherry-based fluorescent reporter for detection of cytochrome *bd* expression in *Mycobacterium marinum*. Cytochrome *bd* was induced by mycolic acid biosynthesis inhibitors such as isoniazid and most prominently by drugs targeting oxidative phosphorylation. We observed no induction by inhibitors of protein-, DNA- or RNA-synthesis. The constructed expression reporter was suitable for monitoring mycobacterial cytochrome *bd* expression during mouse macrophage infection and in a zebrafish embryo infection model when using *Mycobacterium marinum*. Interestingly, in both these infection models cytochrome *bd* levels were considerably higher than during *in vitro* culturing of *M. marinum*. The expression reporter described here can be a valuable tool for elucidating the role of cytochrome *bd* as a survival factor.

## Introduction

In the oxidative phosphorylation pathway the respiratory chain transfers electrons derived from NADH or other reduced shuttle molecules onto oxygen. Coupling redox reactions to proton transport, the respiratory chain complexes establish a proton motive force across a biomembrane, which is subsequently utilized by ATP synthase for synthesis of ATP. The terminal oxidases catalyze the final step in the respiratory chain, the reduction of molecular oxygen. Cytochrome *bd* is such a terminal oxidase and is found in many prokaryotes^1^. Although cytochrome *bd* can contribute to the proton motive force, it is energetically less efficient than other terminal oxidases, such as cytochrome *bo* or cytochrome *aa*_3_-type oxidases^1^. Therefore, cytochrome *bd* typically becomes important under conditions that limit the function of these other terminal oxidases. For instance, cytochrome *bd* is required for respiration under low oxygen tension^2^ and in the presence of nitric oxide^3–5^, peroxynitrite^6^, and hydrogen sulfide^7^ in *Escherichia coli*. Moreover, *E. coli* cytochrome *bd* plays a central role in the defense against hydrogen peroxide^8^ and is strongly induced upon genetic knock-out of ATP synthase^9^. As such, cytochrome *bd* can be regarded as an important component of the bacterial stress response^8^.

In mycobacteria, cytochrome *bd* is found among both fast-growing as well as slow-growing strains. *Mycobacterium tuberculosis* employs a branched respiratory chain, one branch comprises the cytochrome *bc*_1_ complex together with a cytochrome *aa*_3_-type terminal oxidase, while the other branch employs cytochrome *bd* as terminal oxidase^10^. In *M. tuberculosis* and related mycobacterial strains a knock-out of cytochrome *bd* does not influence growth under standard growth conditions^11–13^. However, mycobacterial cytochrome *bd* is upregulated under hypoxia^9^, in the presence of nitric oxide^14^ and during the transition from replicating state to non-replicating persistence^14^. Mycobacterial cytochrome *bd* is also important when the parallel cytochrome *bc*_1_ respiratory chain branch is inactivated^15–18^. In recent years, it has become clear that mycobacterial cytochrome *bd* also plays an important role in survival in the presence of antibiotics. Transcriptional and proteomic studies have revealed that cytochrome *bd* was strongly induced by the ATP synthase inhibitor bedaquiline in *M. tuberculosis* ^19^, and in *Mycobacterium smegmatis* ^20^. Interestingly, *M. tuberculosis* strains lacking cytochrome *bd* displayed enhanced sensitivity for bedaquiline^12^ and cytochrome *bc*_1_ inhibitors^17, 21, 22^. A cytochrome *bd* knock-out in *M. smegmatis* was found hypersensitive for bedaquiline^13, 20^ and for the type-II NADH dehydrogenase effector clofazimine^13^. Clofazimine also induced cytochrome *bd* transcription in *M. tuberculosis* ^23^. These results identified cytochrome *bd* as an important survival factor in the presence of antibiotics and indicate that this enzyme can be an efficient drug target.

To gain insight into the role of cytochrome *bd* as a survival factor it is important to assess which antibacterial drugs and other stressors trigger induction of cytochrome *bd*. To this end, reliable molecular markers are required that are easy to handle and provide information on cytochrome *bd* expression in real-time. Previously, genetic reporters to monitor the expression of the *iniBAC* operon have been constructed and used for characterization of the mycobacterial cell wall stress response upon treatment with ethambutol and isoniazid^24^. These transcriptional fusions of a promoter and a gene encoding a fluorescent reporter proved to be successful in following operon induction kinetics as well as assessment of the mode of action of new drugs (M. Boot *et al*., in preparation). In this study we employ this approach and construct a molecular reporter as a tool for monitoring mycobacterial cytochrome *bd* gene expression in response to antibacterial compounds and during infection.

## Results

### Construction of the cytochrome bd expression reporter

**Figure 1:**
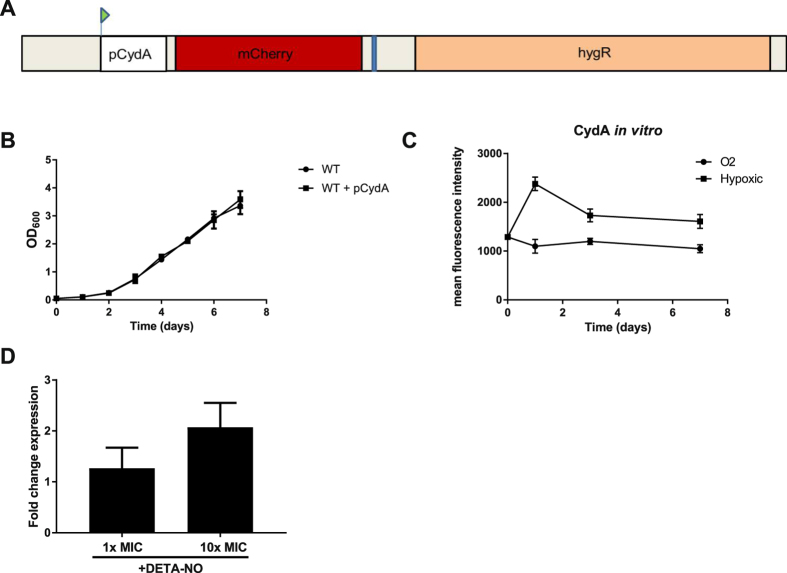
Construction and basic characterization of the cytochrome *bd* expression reporter. (**A**) Genetic construction of the reporter plasmid with the mCherry gene under control of the *cydA* promoter. (**B**) Growth of *Mycobacterium marinum* carrying the *cydA* reporter in 7H9 medium. Three independent biological replicates were monitored for growth. Error bars indicate the standard deviation (s.d.) value. (**C**) mCherry fluorescence emitted by the *cydA* reporter during *in vitro* culture under aerated (normoxia) conditions and hypoxic conditions. Three independent biological replicates were monitored. The fluorescence signal was corrected for autofluorescence of WT bacteria without a plasmid. Error bars indicate the s.d. (**D**) *cydA* induction in response to the nitric oxide donor DETA-NO (1x MIC and 10x MIC) on day 3 after induction. Three independent biological replicates were monitored. Error bars indicate the s.d. value.

The cytochrome *bd* terminal oxidase consists of two main subunits, CydA (subunit I) and CydB (subunit II)^25, 26^. Although the presence of a third, minor subunit has been demonstrated for cytochrome *bd* in various bacterial strains such as *E. coli*.^27, 28^ and *Geobacillus thermodenitrificans* ^29^ our database search indicated no homologues of these minor subunits in the mycobacterial genome. In mycobacteria, the *cydA* and *cydB* genes are located in one operon under control of the cyclic AMP receptor protein^30^. To construct the cytochrome *bd* expression reporter we amplified the region containing the *cydA* promoter (MMAR_2426) from *Mycobacterium marinum* M^USA^. To visualize operon induction, we placed the gene encoding the fluorescent protein mCherry under the control of this promoter (Fig. 1A).

For our study we used *M. marinum*, a slow-growing, pathogenic mycobacterium that is genetically closely related to strains of the *M. tuberculosis* complex^31^. Moreover, *M. marinum* can be used in combination with the zebrafish embryo infection model, a powerful *in vivo* tool that has been shown to mimic the infection processes of *M. tuberculosis* accurately^32, 33^, and can be used for anti-mycobacterial drug discovery^34^.

### Construction and basic characterization of the *cydA* expression reporter

*M. marinum* carrying the plasmid encoding mCherry under control of the *cydA* promoter, referred to as the *cydA* reporter strain, displayed similar growth characteristics as the parent strain (‘wild-type’) (Fig. 1B). We confirmed that the *cydA* reporter strain emitted substantially higher fluorescence than the wild-type control during three days of culture and subsequently deducted the auto-fluorescence of the WT control from induction levels observed in flow cytometry experiments. Next, we assessed the induction of the reporter under normoxia and hypoxia (Fig. 1C). Under normoxia, the observed fluorescence was stable and did not increase over the course of the experiment. When cultured under low oxygen tension, we observed a small *cydA* induction over time (Fig. 1C). In the presence of the nitric oxide donor DETA-NO, previously found to enhance *cydA* transcripts in *M. tuberculosis* ^14^, we observed *cydA* induction at concentrations of 1x MIC (125 uM) and 10x MIC (Fig. 1D). These results indicate that the *cydA* reporter is functional.

### Selected antibacterials in the *M. marinum*/zebrafish embryo infection model

In order to evaluate which type of drugs trigger expression of cytochrome *bd* we first confirmed that selected antibacterials were active against *M. marinum*. Both front-line drugs as well as experimental drugs with various mechanisms of action were tested, including three drugs targeting oxidative phosphorylation (Table 1). The latter included the ATP synthase inhibitor bedaquiline (BDQ)^35–37^, the cytochrome *bc*_1_ inhibitor Q203^38^ and the type-II NADH dehydrogenase effector clofazimine (CFZ)^39^. BDQ has been approved for treatment of multi-drug resistant tuberculosis, Q203 presently is evaluated in phase I clinical trials, and clofazimine is already in clinical use for treatment of leprosy and presently is being re-purposed as anti-TB drug.

**Table 1:** List of antibiotics and minimal inhibitory concentrations (MICs) used in this study.

Inhibitor	Target pathway	MIC (*M. marinum*)
Isoniazide (INH)	Cell wall synthesis	10 µg/mL^24^
Ethambutol (EMB)	Cell wall synthesis	1 µg/mL^24^
Rifampicin (RIF)	RNA synthesis	0.32 µg/mL^24^
Streptomycin (STR)	Protein synthesis	4 µg/mL^24^
Ciprofloxacin (CIP)	DNA synthesis	4 µg/mL^24^
Bedaquiline (BDQ)	Oxidative phosphorylation	0.062 μM (this study)
Q203	Oxidative phosphorylation	3.5 μM^38^
Clofazimine (CFZ)	Oxidative phosphorylation	0.8 μg/mL (this study)

**Figure 2:**
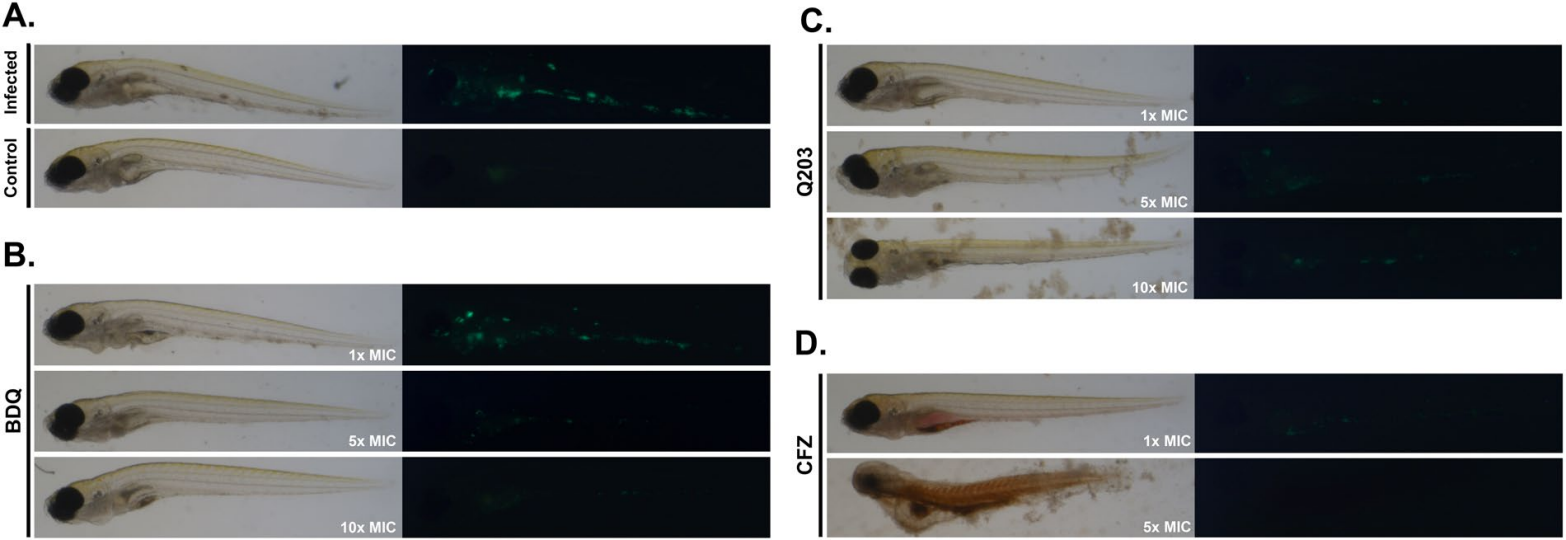
Selected drugs are active in a zebrafish embryo infection model 3 days post treatment. (**A**) Infected fish, untreated (top) brightfield and fluorescence image as compared to a control, uninfected fish. (**B**) BDQ treated fish. (**C**) Treatment with Q203. (**D**) treatment with CFZ. Zebrafish infection experiments were performed with at least three biological replicates.

We determined the minimal inhibitory concentrations (Table 1) and also characterized BDQ, Q203 and CFZ activity against *M. marinum* in a zebrafish embryo infection model. The zebrafish embryo infection model allows for real-time visualization of fluorescently labeled pathogens^40, 41^. The colonization of zebrafish embryos by *M. marinum* is visualized by a constitutively expressed mEos3.1 variant and can be quantified by fluorescence microscopy^40, 42^ (Fig. 2A). BDQ prevented bacterial colonization in a dose-dependent manner (Fig. 2B). Q203 also suppressed bacterial growth at 1x MIC, however, at 5x MIC and 10x MIC the compound seemed to precipitate, apparently limiting its activity (Fig. 2C). At 1x MIC, CFZ cleared bacterial loads, whereas concentrations of 5x MIC and higher were toxic for the zebrafish embryo host under these conditions (Fig. 2D). These results show that BDQ, Q203 and CFZ are active against *M. marinum*.

### Cytochrome bd induction by antibacterial compounds

**Figure 3:**
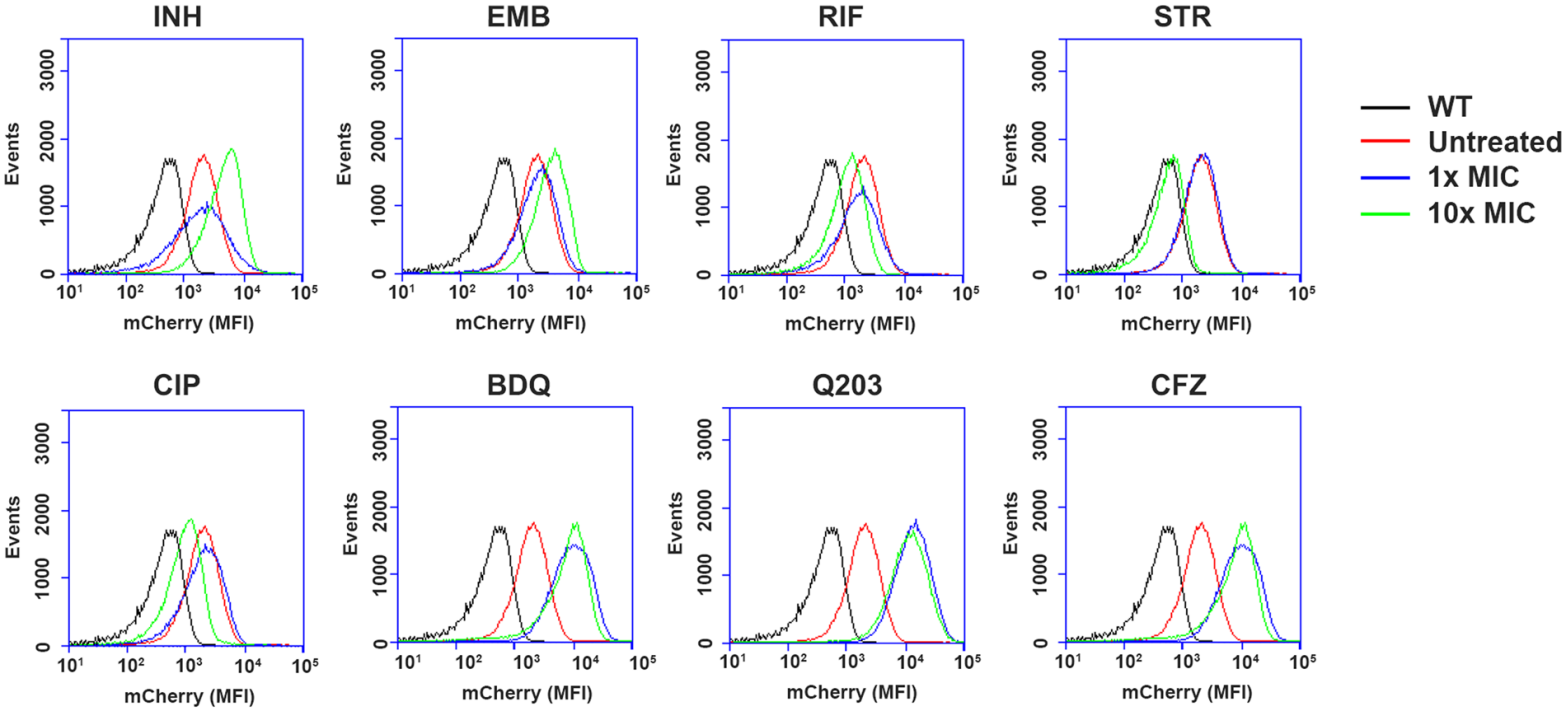
Differential regulation of *cydA* in response to antibacterials analyzed by flow cytometry. A histogram plot showing the number of events (y-axis). Peak height indicates mCherry fluorescence intensity (x-axis) for: Untreated samples (red line), 1x MIC of the shown antibiotic (blue line) and 10x MIC (green line). WT without *cydA* reporter served as a background control (black line). Three independent experiments were performed, each histogram represents data from one representative experiment with 30,000 cells.

**Figure 4:**
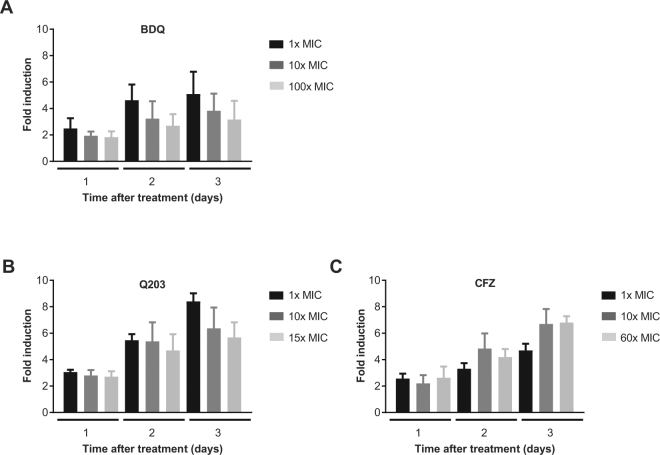
Time- and concentration dependency of *cydA* induction by inhibitors of oxidative phosphorylation. (**A**) Response to BDQ for 1x, 10x and 100x MIC over time. (**B**) Response to Q203 for 1x, 10x and 15x MIC. (**C**) Response to CFZ for 1x, 10x and 60x MIC. The fold inductions were calculated by dividing the mean fluorescence intensities of the treated samples by the corresponding untreated controls. The data of three independent biological replicates were used. Error bars represent the s.d. values.

Ethambutol and isoniazid, both inhibitors of mycobacterial cell envelope biosynthesis^43, 44^, showed slight *cydA* induction when high concentrations were applied (10x MIC) (Fig. 3). In contrast, the RNA polymerase inhibitor rifampicin decreased *cydA* expression at 10x MIC as compared to control samples (Fig. 3). Among the second-line drugs used for tuberculosis treatment, pronounced *cydA* downregulation was observed for the protein synthesis inhibitor streptomycin at 10x MIC (Fig. 3), and for the DNA gyrase inhibitor ciprofloxacin at 10x MIC (Fig. 3). Next, we tested drugs active on three distinct targets in the oxidative phosphorylation pathway. As depicted in Fig. 4, all three drugs, BDQ, CFZ and Q203, significantly induced cytochrome *bd* expression at 1x MIC and 10x MIC (Fig. 3). These data reveal that inhibitors with different mechanisms of action can induce *cydA* expression, but induction was most prominent in response to inhibitors of oxidative phosphorylation. We confirmed the validity of the *cydA* reporter by quantitative reverse-transcription PCR, which clearly showed increased levels of *cydA* transcripts in the presence of inhibitors of oxidative phosphorylation (Supplementary Figure 1). As these three drugs are slow-acting and display only minimal kill of *M. tuberculosis* in the first 2–3 days of treatment^19, 38, 45^, we subsequently investigated time- and concentration dependency of *cydA* expression in more detail for these drugs.

### Time- and concentration dependency of *cydA* induction

For the ATP synthase inhibitor BDQ, cytochrome *bd* induction increased from 2.3 fold at day 1 to 4.5-fold at day 2 of treatment, with only marginal changes between day 2 and day 3 time points (Fig. 4A). For this drug, maximal induction was achieved at 1x MIC and induction dropped at elevated concentrations (10x MIC and 100x MIC) at all three time points investigated (Fig. 4A). For the cytochrome *bc*_1_ inhibitor Q203 *cydA* induction levels at 1x MIC induction steadily increased to 8.3-fold at day 3 (Fig. 4B). Similar to BDQ, induction by Q203 at 1x MIC was stronger as compared to higher drug concentrations at all three time points (Fig. 4B). The NDH-2 effector CFZ at 1x MIC triggered slowly increasing *cydA* levels until day 3 (Fig. 4C). CFZ displayed only marginal concentration-dependency of induction at day 1 and day 2, whereas at day 3 induction was clearly higher at 10x MIC (6.6-fold) and 60x MIC (6.9-fold (Fig. 4C). For comparison, the time- and concentration dependency of *cydA* regulation by the front-line drugs are shown in Supplementary Figure 2.

In line with the delayed bactericidal activity of the three oxidative phosphorylation inhibitors induction of cytochrome *bd* was time-dependent and maximal induction was not reached before two days of treatment. Both BDQ and Q203 showed maximal cytochrome *bd* expression at concentrations of 1x MIC, whereas the higher, bactericidal concentrations were less effective in cytochrome *bd* induction. These two inhibitors strongly decrease bacterial ATP levels^19, 36, 38^. It can be speculated that pronounced ATP depletion at elevated drug concentrations renders the bacteria less capable of mobilizing an effective metabolic response.

### The *cydA* reporter induced in macrophages

**Figure 5:**
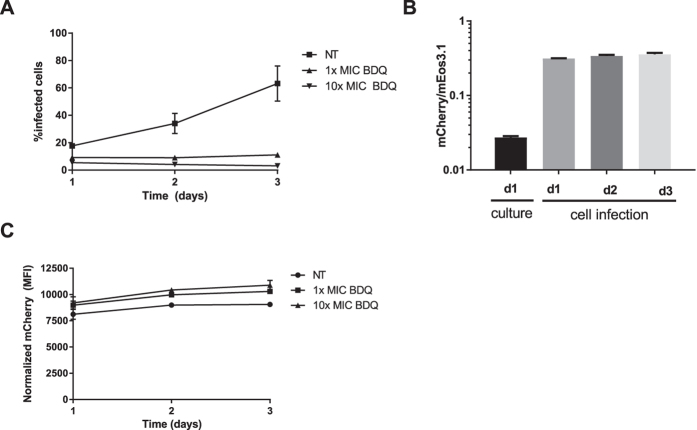
The *cydA* reporter in a mouse macrophage infection model. (**A**) The percentage of infected RAW cells over time for NT (non-treated) as compared to cells that were treated with 1x or 10x MIC BDQ. The experiment was performed in triplicate and error bars represent s.d. values. (**B**) Flow cytometry experiment determining the ratio of mCherry signal (MFI) relative to the mEos3.1 signal (MFI) for bacteria in culture compared to values obtained from mouse macrophage cell infection. Data represents triplicate measurements and error bars represent s.d. values. (**C**) Normalized mCherry MFI over time during cell infection, comparing non-treated (NT) to BDQ-treated infected macrophages. The experiment was performed in triplicate and s.d. values are indicated by the error bars.

To evaluate if cytochrome *bd* expression can be monitored under conditions as encountered within a mammalian host we examined the *cydA* reporter in a mouse macrophage infection model. To differentiate between infected and non-infected macrophages and to assess which proportion of the infected cells expressed the *cydA* reporter, we introduced an integrative plasmid with a gene encoding fluorescent protein mEos3.1 under control of the *hsp60* promoter in our *M. marinum cydA* reporter strain. Expression levels of mCherry (*cydA*) and mEos3.1 were assessed by flow cytometry using a gating strategy depicted in Supplementary Figure 3. The analysis showed that the percentage of untreated mEos3.1-positive RAW macrophage cells steadily increased within a 3-day period after start of infection (Fig. 5A). Treatment with BDQ suppressed infection (Fig. 5A). To assess *cydA* expression relative to bacterial cell number, we normalized the mCherry signal relative to the mEos3.1 signal. Whereas for *M. marinum* in culture the mCherry signal was ~70fold lower than the mEos3.1 signal, during RAW cell infection the ratio mCherry/mEos3.1 ratio increased to ~1:3 within one day. Prolonged incubation resulted in a marginal further increase between day 1 and day 3 of infection (Fig. 5B). Upon treatment with BDQ an additional increase of the normalized *cydA* expression in the RAW cell infection model was observed (Fig. 5C). These results suggest that the *cydA* reporter is working in a mouse cell line infection model and that cytochrome *bd* is induced during intracellular growth.

### The *cydA* reporter is functional in a vertebrate host system

**Figure 6:**
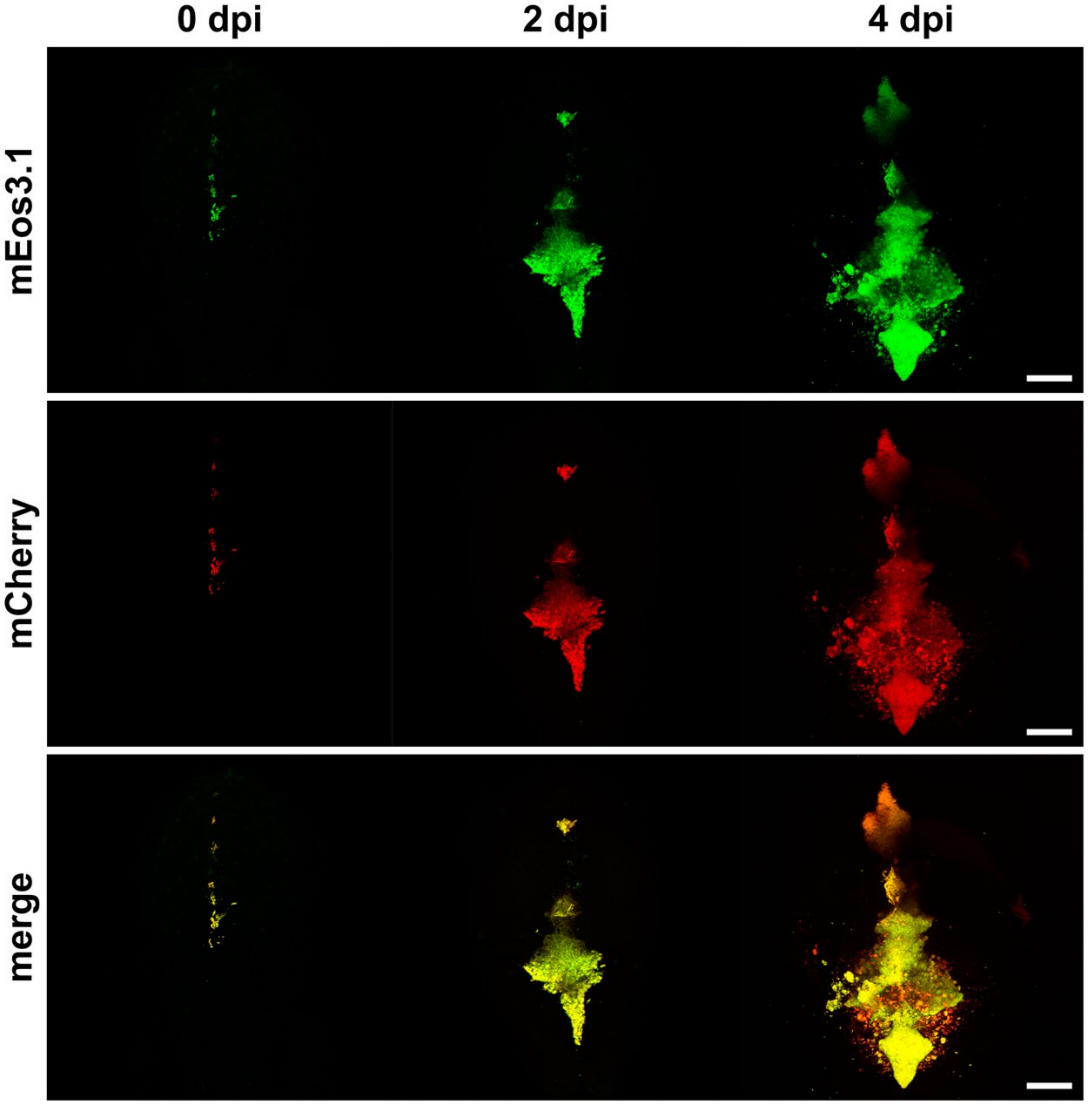
The *cydA* reporter in a zebrafish embryo model. Confocal microscopy images of casper zebrafish embryos at maximum projection. Time course of mEos3.1 and mCherry signals during infection (dpi = days post infection). Upper row: mEos3.1 signal indicates infection. Middle row; mCherry fluorescence indicates *cydA* transcription. Lower row: overlay of upper and middle row. Scale bars, 100 µm. Three independent experiment were performed, the images were selected from a representative experiment.

Next, we explored if the constructed *cydA* reporter can also be used to study cytochrome *bd* expression in a zebrafish embryo infection model. *M. marinum* carrying the *cydA* reporter and the constitutive mEos3.1 marker were injected into in the hindbrain ventricle of zebrafish embryos. Both mCherry and mEos3.1 fluorescence signals were readily detectable by confocal microcopy. The mEos3.1 signal allowed for monitoring the spread of the infection within the hindbrain (Fig. 6, upper row). Treatment with BDQ decreased bacterial growth (Supplementary Figure 4), supporting the usage of a hindbrain ventricle infection model to address drug efficacy. Although quantification of *in vivo* fluorescent signals is difficult, a similar trend was observed for the mCherry signal as in the macrophage infection model. Even without antibiotic treatment high induction of the mCherry reporter was visible *in vivo*, exceptionally pronounced at day 4 post infection (Fig. 6, middle and lower row). Due to the high *cydA* expression levels in the non-treated control we were not able to quantify if treatment with BDQ additionally induced *cydA* (Supplementary Figure 4). Interestingly, in certain infected areas of the central nervous system mCherry induction appeared exceptionally strong (Fig. 6, lower row), which may be due to varying levels of cytochrome *bd* inducing factors in specific micro-environments. These results show that the *cydA* reporter allows for monitoring cytochrome *bd* expression *in vivo* and indicates that cytochrome *bd* is produced at elevated levels during infection in a vertebrate host.

## Discussion

Previously, alkaline phosphatase fusions to subunit I or subunit II have been applied to investigate cytochrome *bd* topology^46^ and a galactosidase-based marker was used to assess cytochrome *bd* expression under hypoxia^11^. Green Fluorescent Protein (GFP)-fusions to subunit II facilitated investigation of cytochrome *bd* dynamics in the *E. coli* cytoplasmic membrane^47^ and in *Corynebacterium glutamicum* GFP under the control of the cytochrome *bd* promoter was used to study expressional control of the branched respiratory chain^48^. In our study, we describe an expression reporter for cytochrome *bd* in a pathogenic (myco)bacterium. *M. marinum*, and we applied this reporter to evaluate differential regulation of cytochrome *bd* in response to antibiotics and during infection. The marker revealed cytochrome *bd* induction in response to antibacterials of different classes, but in particular to inhibitors of oxidative phosphorylation. Drugs targeting this pathway are under consideration as components for next-generation anti-tuberculosis chemotherapy regimen^49–52^. The reporter constructed here may assist in characterizing new candidate antibacterials and may provide input for the rational combination of antibacterials in drug regimen. It would be interesting to test if induction of cytochrome *bd* in response to inhibition of oxidative phosphorylation is also found in non-mycobacterial pathogens, such as *Staphylococcus aureus*, *Streptococcus pneumoniae* or *Acinetobacter baumanii*, for which small molecules targeting ATP synthase and components of the respiratory chain have been reported previously^53–55^.

Induction of cytochrome *bd* in response to clofazimine, a ROS-producing drug^39^, is most likely due to this enzyme’s ability to metabolize and/or prevent the formation of peroxides. Cytochrome *bd* as a high-affinity oxygen scavenger^2^ may decrease the intracellular oxygen tension, thereby preventing the formation of reactive oxygen species. Moreover, for cytochrome *bd* from *E. coli* both catalase activity^56^ and peroxidase activity^57^ has been reported. This enzyme may thus directly metabolize peroxides. Activity of BDQ and Q203 was not found linked to ROS production^13, 18^. Induction of cytochrome *bd* may facilitate respiratory electron flow when the cytochrome *bc*_1_ complex is inhibited by Q203. Enhanced levels of cytochrome *bd* in response to the ATP synthase inhibitor BDQ may prevent membrane hyperpolarization and/or maintain respiratory electron flow. Alternatively, cytochrome *bd* induction may be associated with the enhanced respiratory flux and the uncoupling effect reported for bactericidal concentrations of BDQ^20, 58^.

The *cydA* reporter revealed high cytochrome *bd* levels for *M. marinum* in two relevant infection models, illustrating the importance of this respiratory protein for survival in the host. In a recent report no clear attenuation phenotype was found for a Δ*cydAB* strain in mice^22^, however, consistent with our findings, earlier results indicated that genetic inactivation of cytochrome *bd* in *M. tuberculosis* decreased bacterial counts in a mouse infection model^14^. In addition, numerous studies suggest that lack of a functional cytochrome *bd* severely compromises bacterial viability during infection for a broad spectrum of Gram-positive and Gram-negative bacterial strains, such as Group B *Streptococcus* ^59^, *Staphylococcus aureus* ^60^, *Shigella flexneri* ^61^, *Brucella abortus* ^62^, *Salmonella enterica* ^63, 64^ and uropathogenic *E. coli* strains^65^. It needs to be clarified to which extent elevated cytochrome *bd* levels represent a general feature of pathogens residing in a vertebrate host. Fluorescent expression reporters, such as constructed and characterized here, may be important tools for elucidating the role of cytochrome *bd* in pathogenic bacteria.

## Materials and Methods

### Construction of the *cydA* reporter plasmid

A list of primers can be found in supplemental Table 1. Anchored primers containing either an XbaI site or BamHI site were used to amplify a 290-bp fragment that included the *cydA* promoter from *M. marinum* M^USA^ genomic DNA. Subsequently, both the pAL5000 plasmid derivative pSMT3-iniB-mCherry^24^ and the amplified PCR product were digested with XbaI and BamHI, mixed, and ligated, resulting in pSMT3-pCydA-mCherry.

### Strains and culturing conditions

*M. marinum* wild type M^USA^ ^66^ was used as a basis for mycobacterial experiments. Cultures were grown in Middlebrook 7H9 liquid medium supplemented with Middlebrook ADC (Difco) and 0.05% Tween 80. Antibiotics (Sigma) were added to cultures where indicated. Antibiotics for the induction experiments were routinely added to log-phase cultures at the indicated final concentrations. Middlebrook 7H10 solid agar supplemented with Middlebrook OADC was used for bacterial culturing on agar plates. Both cultures and plates were grown at 30 °C. For hypoxic conditions, *M. marinum* cultures were grown to log phase in a culture flask in Middlebrook 7H9 medium at 30 °C and diluted to an OD600 of 0.3 per mL. A total of 1.5 mL was incubated per screwcap tube to acquire a headspace ratio (HSR) of ~0.5^67^. The tubes were incubated at 30 °C on a shaker. Per time point one tube was opened and the hypoxic culture was tested for growth, as measured by OD600, and expression *of cydA* by flow cytometry. Experiments were performed three independent times

### Flow cytometry analysis

The induction of *cydA* was measured by flow cytometry. *M. marinum* containing the reporter plasmid was grown to an OD600 of 0.2–0.3, and antibiotics were added to the indicated concentrations for a specified amount of time. For each time point, 1 mL culture was collected, spun down by centrifugation, washed once with PBS containing 0.05% Tween 80 and resuspended in 100 µl 0.05% Tween 80. Cell infections were also analyzed by flow cytometry. Cell infection samples were resuspended in PBS. Samples were acquired on a BD Accuri C6 flow cytometer (BD Biosciences) equipped with a 488-nm laser and 610/20-nm filter to detect mCherry and 530/30-nm filter to detect mEos3.1. The exact gating strategy varied between experiments. Per sample, 30,000 gated events were collected per time point, and data were analyzed using BD CFlow software and GraphPad prism 6.

### RNA isolation and qRT-PCR

RNA isolation was performed with a NucleoSpin® RNA kit (Machery Nagel). In total 25 OD units were spun down and used for isolation per sample. Isolation was performed with three biological replicates. There was one deviation compared to the first step of the manufacturer’s protocol to lyse the mycobacterial cells. Cells were beadbeated in 500 μL Buffer RA1 and 5 μL ß-mercaptoethanol for 1 minute. Subsequent steps were according to the protocol provided by the manufacturer. RNA samples were subsequently treated with DNAseI (Thermo Scientific) according to manufacturer’s protocol. Equal amounts of RNA were analyzed in an ABI7500 thermocycler using a EXPRESS One-Step Superscript® qRT-PCR Kit. Instructions supplied with the kit were followed. *sigA* was used as a reference gene for qRT-PCR. The sequences for the primers and probes can be found in Supplementary Table 1. The resulting data of triplicates were analyzed using the ABI7500 software.

### Cell infection assay with RAW macrophages

For RAW cell infection, 2 × 10^4^ cells per well were seeded in 12 wells plates (Corning) and grown for 4 days until ~70% confluence. The *cydA* reporter strain that also contained the integrated *pMV-hsp60-mEos3.1* was grown to an OD600 of 0.8–1.5 and washed with DMEM + FBS (10%) prior to infection. Bacterial cells were subsequently added to a multiplicity of infection of 2. RAW cells were incubated at 33 °C After three hours, extracellular mycobacteria were removed by washing 2 times with PBS. Amikacin was added to a final concentration of 200 μg/mL for 3 hours to kill any remaining extracellular mycobacterial cells. Cell were then washed and BDQ was added at the indicated concentrations. At time points 24 hpi, 48 hpi, and 72 hpi RAW cells were harvested by addition of trypsine (Sigma). The trypsin was neutralized by addition of DMEM + FBS (10%) and cells were pelleted at 1400 rpm. Cells were subsequently fixated with 4% PFA for 30 minutes at room temperature, washed with PBS and analyzed by flow cytometry.

### Infection of zebrafish embryos

To assess the antimycobacterial effect of Q203, clofazimine and BDQ, zebrafish embryos were infected with 100 colony forming units (CFUs) in the caudal vein by microinjection with *M. marinum* M^USA^ containing the plasmid *pSMT3-hsp60-mEos3.1*. (Stoop *et al*./van Leeuwen *et al*. unpublished). The embryos were treated 1 day post infection with the aforementioned antibiotics at the indicated concentrations. Fluorescence was monitored at specific time-points with a Leica MZ16FA fluorescence microscope and a Leica DFC420C camera. All experiments were performed at least three times. For expression analysis in zebrafish embryos *pMV-hsp60-mEos3.1* ^68^ was introduced in *M. marinum* M^USA^ already containing the cydA reporter plasmid. Zebrafish embryos were injected with 2000 CFUs by microinjection in the hindbrain ventricle, as previously described^24, 41^. Subsequently, zebrafish embryos were fixated O/N at 0, 2 and 4 days post infection in 4% paraformaldehyde dissolved in PBS, washed with PBS and subsequently stored in PBS at 4 °C. For confocal microscopy, zebrafish were mounted in 1% low-melting-point agarose in PBS and imaged with a Leica TCS SP8 confocal microscope.

### Animal experiments

All methods were carried out in accordance with relevant guidelines and regulations. *Danio rerio* (zebrafish) were handled in compliance with the local animal welfare regulations and maintained according to standard protocols (zfin.org). The breeding of adult fish was approved by the local animal welfare committee (Animal Experimental licensing Committee, DEC) of the VU University medical center. All protocols adhered to the international guidelines specified by the EU Animal Protection Directive 86/609/EEC, which allows zebrafish embryos to be used up to the moment of free-living (approximately 5–7 days after fertilization). Because embryos used in this study met these criteria, this specific study was therefore approved by the Animal Experimental Licensing Committee of the VU University medical center (Amsterdam, the Netherlands).

### Data availability

All data generated or analysed during this study are included in this published article or its Supplementary Information files.

## Electronic supplementary material

Supplementary file containing all supplementary data
