# Supplementary material for: A fluorescence-based reporter for monitoring expression of mycobacterial cytochrome bd in response to antibacterials and during infection
Source: Sci Rep. 2017 Sep 6;7:10665. doi: 10.1038/s41598-017-10944-4 (PMC5587683; doi:10.1038/s41598-017-10944-4)
Supplement: Supplementary file 1 — Supplementary file containing all supplementary data [file 41598_2017_10944_MOESM1_ESM.doc]

**A fluorescence-based reporter for monitoring expression**

**of mycobacterial cytochrome *bd***

**in response to antibacterials and during infection.**

Maikel Boot^1^, Kin Ki Jim^1^, Ting Liu^2^, Susanna Commandeur^1^, Ping Lu^2^, Theo Verboom^1^, Holger Lill^2^, Wilbert Bitter^1,2^, Dirk Bald^2*^

^1^Department of Medical Microbiology and Infection Control, VU University Medical Center, De Boelelaan 1108, 1081 HZ Amsterdam, The Netherlands.

^2^Department of Molecular Cell Biology, Amsterdam Institute for Molecules, Medicines and Systems, Faculty of Earth- and Life Sciences, Vrije Universiteit Amsterdam, De Boelelaan 1108, 1081 HZ Amsterdam, The Netherlands.

**Supplementary Figure 1**: qRT-PCR to confirm upregulation of *cydA* in *M. marinum.*
qRT-PCR was utilized to measure induction the *cydA* reporter after three days of treatment with BDQ, Q203, CFZ and INH (1x MIC each), as compared to a not treated control. Values are from three biological replicates, error bars indicate the standard deviation (s.d.) values.

**
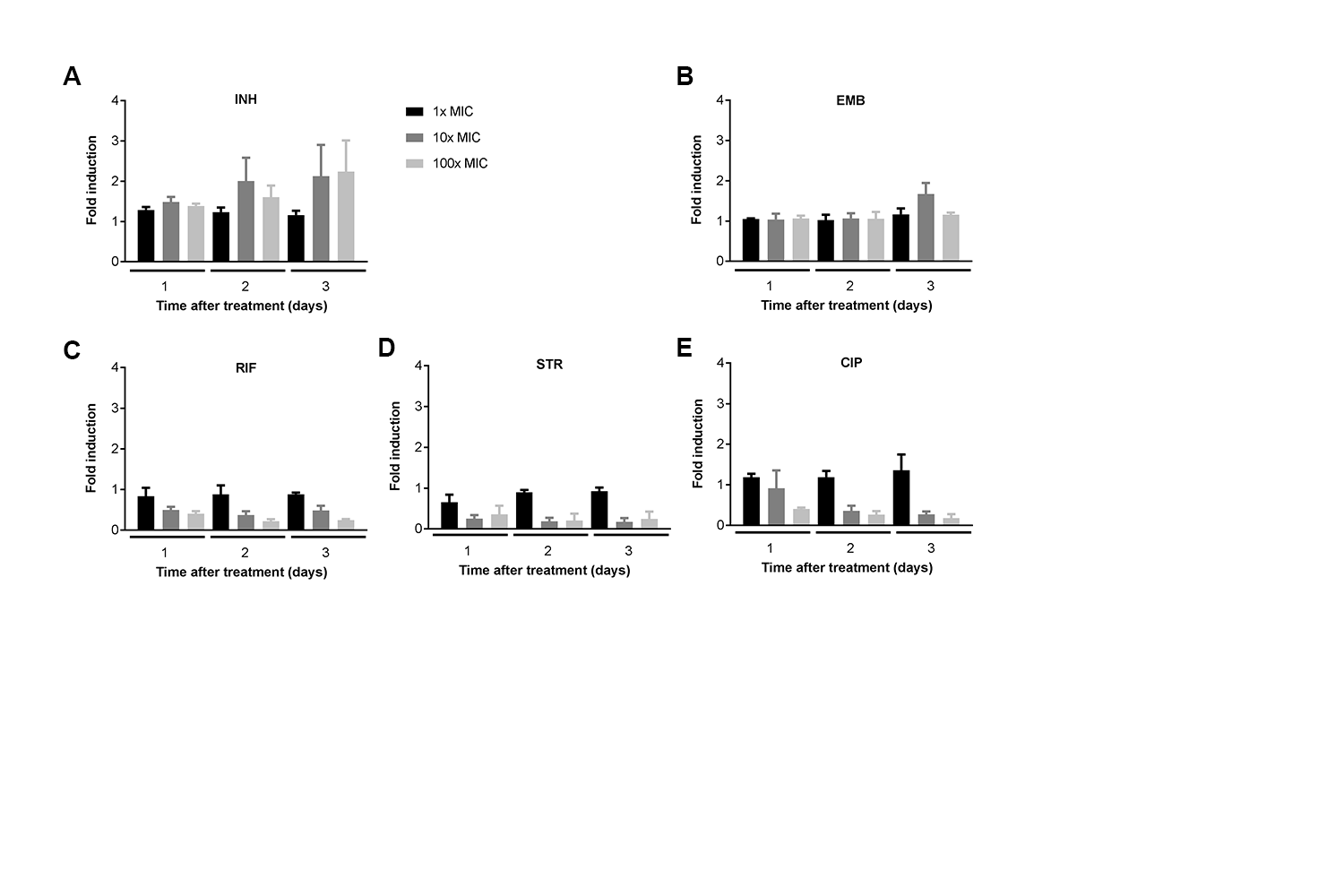
**

**Supplementary Figure 2**: Time- and concentration dependency of *cydA* induction by other first and second-line antibiotic for 1x, 10x and 100x MIC over time **A**: Response to isoniazid. **B**: Response to ethambutol. **C**: Response to rifampicin. **D**: Response to streptomycin **E**: ciprofloxacin. Three independent experiments were performed. Error bars indicate s.d. values.

**Supplementary Figure 3**: The gating strategy for RAW cell infection
**A**: Gating of the intact RAW cell population after infection (P1). **B**: A non-infected control was used to set gate R2 in P1 and defined as positive for mEos3.1. **C**: Cells that fall in gate R2 were used for analysis of mCherry signal.


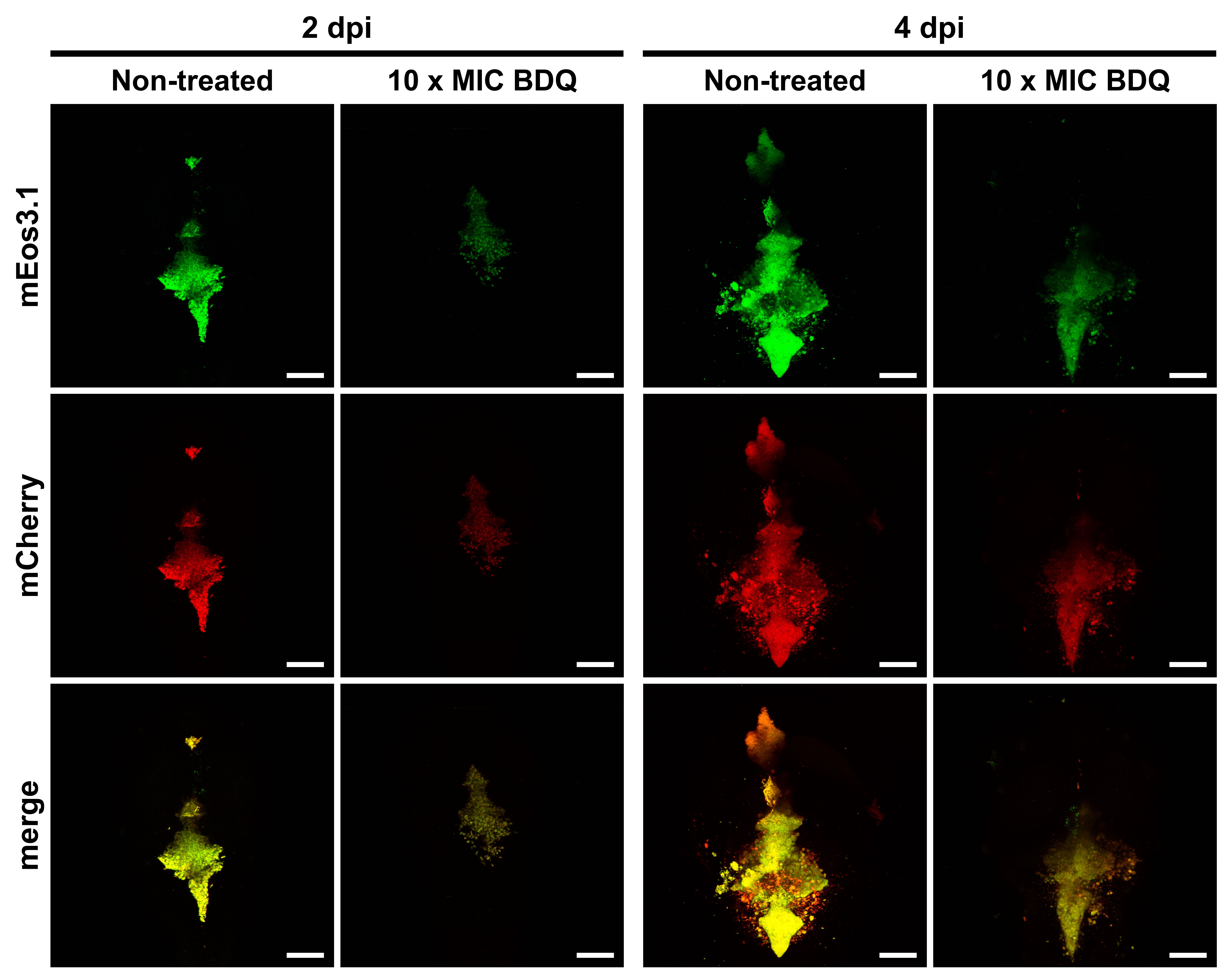


**Supplementary Figure 4**:

BDQ decreases bacterial growth in the zebrafish hindbrain infection. Treatment of zebrafish embryos was started directly after infection. After 2dpi and 4dpi a significant reduction in bacterial load can be seen.

**Supplementary Table 1:** Primers and probes used in this study.
